# Associação entre a Relação Neutrófilo-Colesterol HDL e Infarto do Miocárdio: Um Estudo Transversal

**DOI:** 10.36660/abc.20250718

**Published:** 2026-08-24

**Authors:** Youpeng Wang, Qian Yin, Ying Lei, Juncang Duan

**Affiliations:** 1 Affiliated Jinhua Hospital Zhejiang University School of Medicine Jinhua China Department of Cardiology – Second District – Affiliated Jinhua Hospital, Zhejiang University School of Medicine, Jinhua – China

**Keywords:** Infarto do Miocárdio, Neutrófilos, HDL-Colesterol, Inquéritos Nutricionais

## Abstract

**Fundamento:**

O infarto do miocárdio (IM) continua sendo um importante problema de saúde global. Os papéis da inflamação e do metabolismo lipídico na patogênese do IM têm atraído mais atenção. O IM permanece como um importante problema de saúde global. O papel da inflamação e do metabolismo lipídico na patogênese do IM tem despertado crescente interesse. A razão entre neutrófilos e colesterol de lipoproteína de alta densidade (NHR) é um novo biomarcador composto que integra marcadores de inflamação e do metabolismo lipídico, mas sua associação com o IM não está clara.

**Objetivos:**

Investigamos a associação entre NHR e IM.

**Métodos:**

Analisamos dados de 25.248 participantes elegíveis do NHANES entre 2005 e 2016. O histórico de IM foi determinado por meio de questionários, e os valores de NHR foram obtidos a partir de exames laboratoriais. Modelos de regressão logística foram utilizados para avaliar as associações entre NHR, contagem de neutrófilos e colesterol de lipoproteína de alta densidade (HDL-C) com as probabilidades de IM. A análise de spline cúbica restrita (SCR) foi utilizada para avaliar possíveis associações não lineares. Análises de subgrupos e testes de interação foram realizados para explorar a heterogeneidade potencial entre subgrupos demográficos e clínicos. Análises de sensibilidade foram realizadas para avaliar a robustez dos principais achados. Um valor de p bicaudal <0,05 foi considerado estatisticamente significativo.

**Resultados:**

Níveis mais elevados de NHR foram associados independentemente a maiores chances de IM. No modelo totalmente ajustado, os participantes no quartil mais alto de NHR apresentaram 49% mais chances de IM do que aqueles no quartil mais baixo. A contagem de neutrófilos isoladamente não apresentou associação significativa com IM, enquanto os níveis de HDL-C apresentaram associação inversa com IM. A SCR sugeriu uma associação não linear modesta entre NHR e IM. Embora um ponto de inflexão baseado em dados tenha sido identificado em 6,93, esse resultado deve ser interpretado como exploratório e descritivo, e não como um limiar clinicamente significativo. Análises de subgrupos sugeriram uma associação mais forte em mulheres, e a interação por sexo foi estatisticamente significativa. Análises de sensibilidade confirmaram a robustez desses achados.

**Conclusão:**

Nesta amostra ampla e representativa, níveis elevados de NHR foram associados de forma independente a maiores chances de IM, sugerindo que a NHR pode servir como um marcador epidemiológico potencialmente informativo para IM. Estudos prospectivos são necessários para determinar a causalidade e elucidar os mecanismos subjacentes.

## Graphical abstract

**Figura Central:**
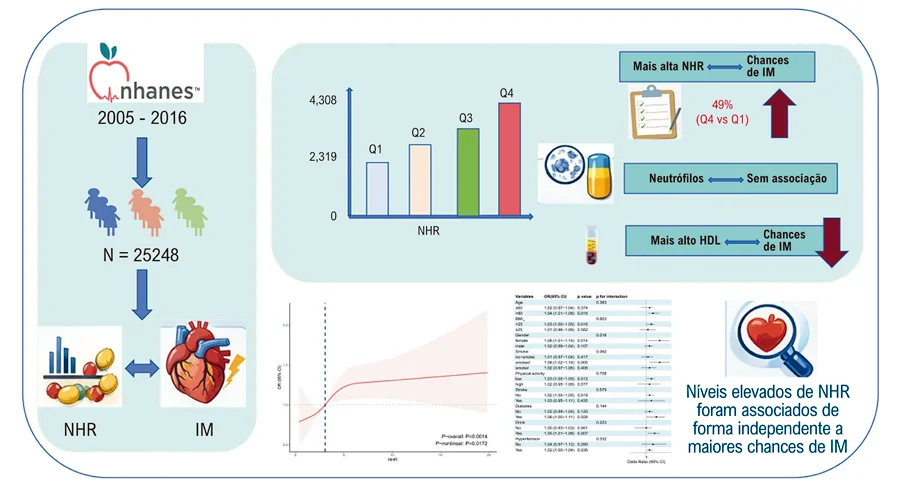
Associação entre a Relação Neutrófilo-Colesterol HDL e Infarto do Miocárdio: Um Estudo Transversal

## Introdução

O infarto do miocárdio (IM) continua sendo uma das principais causas de morbidade e mortalidade cardiovascular, impondo uma carga substancial à saúde pública global.^1^ De acordo com o Estudo Global da Carga de Doenças, a doença cardíaca isquêmica causou 9,14 milhões de mortes em todo o mundo em 2019, representando 49,2% de todas as mortes relacionadas a doenças cardiovasculares, e a carga da doença cardíaca isquêmica continua a aumentar.^2^ O IM impõe uma pressão considerável sobre os sistemas de saúde e as famílias, contribuindo substancialmente para a mortalidade prematura.^3^ Portanto, identificar os fatores associados ao IM é essencial para fundamentar estratégias eficazes de prevenção precoce.

Estudos recentes demonstraram que as células inflamatórias desempenham um papel fundamental na progressão do IM. A infiltração excessiva de neutrófilos e linfócitos, juntamente com a ativação anormal do sistema complemento, exacerba significativamente a lesão miocárdica. Além disso, essas células e as citocinas e quimiocinas que secretam regulam coletivamente o reparo e a remodelação miocárdica, e a desregulação desses processos é um dos principais fatores que contribuem para a remodelação ventricular adversa e a insuficiência cardíaca.^4,5^ O metabolismo lipídico também está intimamente associado ao IM. Especificamente, os ácidos graxos poli-insaturados ômega-3 de cadeia longa e o ácido esteárico apresentam associação inversa com o risco de IM, enquanto o ácido araquidônico apresenta correlação positiva.^6^ Além disso, níveis elevados de lipoproteínas de densidade muito baixa, lipoproteínas de baixa densidade e seu conteúdo de colesterol, bem como triglicerídeos, estão associados a um risco aumentado de IM, enquanto o colesterol da lipoproteína de alta densidade (HDL-C) de partículas grandes está inversamente associado ao risco de IM.^7^

Tanto a inflamação quanto a dislipidemia são fatores-chave na patogênese do IM.^8^ A razão entre neutrófilos e colesterol HDL (NHR) é um novo biomarcador composto que integra essas duas vias e pode refletir interações sinérgicas não capturadas por biomarcadores individuais. Os neutrófilos infiltram o miocárdio infartado e liberam citocinas pró-inflamatórias e proteases que exacerbam a lesão miocárdica. Em contraste, o HDL-C exerce efeitos cardioprotetores ao inibir a ativação do fator nuclear kappa B (NF-κB), modular as respostas de citocinas inflamatórias e promover o transporte reverso de colesterol.^9-12^ Ao integrar essas duas vias, a NHR pode refletir o equilíbrio líquido entre os mecanismos pró-inflamatórios e protetores envolvidos no IM. Estudos anteriores demonstraram a utilidade da NHR na avaliação de anormalidades metabólicas no diabetes tipo 2,^13^ prevendo a estabilidade da placa aterosclerótica^14^ e avaliando o prognóstico da sepse.^15^ Além disso, a NHR tem sido associada a desfechos cardiovasculares adversos, incluindo doença arterial coronariana e vulnerabilidade da placa na aterosclerose.^16-19^ Embora a NHR ainda não tenha sido amplamente adotada na prática clínica de rotina, sua derivação de medições laboratoriais padrão a torna um biomarcador conveniente e potencialmente prática para a avaliação do risco cardiovascular.

No entanto, a relação entre NHR e IM não foi totalmente elucidada em grandes populações representativas a nível nacional. Embora a NHR tenha sido investigada em outros contextos cardiovasculares e metabólicos, sua associação específica com o IM permanece incompletamente caracterizada. Para preencher essa lacuna, analisamos dados do *National Health and Nutrition Examination Survey* (NHANES) para avaliar se uma NHR mais elevada está associada a um aumento da probabilidade de IM em adultos nos EUA. Nossa hipótese era de que uma NHR elevada está associada a uma maior probabilidade de IM após o ajuste para fatores de risco cardiovascular estabelecidos, e que essas associações podem variar de acordo com o sexo e a idade. Essas análises têm como objetivo examinar a relevância epidemiológica da NHR e gerar hipóteses para futuros estudos prospectivos e mecanísticos.

## Materiais e métodos

### Fonte de dados e população do estudo

O NHANES é um programa de vigilância em saúde com representatividade nacional, conduzido pelo Centro Nacional de Estatísticas de Saúde (NCHS) sob os auspícios dos Centros de Controle e Prevenção de Doenças dos EUA (CDC).^20^ O levantamento integra entrevistas estruturadas, exames físicos e testes laboratoriais para coletar dados abrangentes de saúde e nutrição da população civil não institucionalizada dos Estados Unidos, permitindo assim o monitoramento do estado de saúde nacional, dos níveis nutricionais e das tendências de prevalência de doenças. Todos os instrumentos e protocolos do levantamento são padronizados e documentados publicamente pelo NCHS. Este estudo transversal foi relatado de acordo com as diretrizes do *Strengthening the Reporting of Observational Studies in Epidemiology* (STROBE) (Tabela Suplementar 1).^21^

Neste estudo, utilizamos dados dos ciclos do NHANES entre 2005 e 2016, que inicialmente incluíam 60,936 participantes. Excluímos indivíduos com menos de 20 anos de idade (n = 26.756), gestantes (n = 523), com dados faltantes na NHR (n = 3.358), com dados faltantes no IM (n = 45) e aqueles com informações faltantes nas covariáveis (n = 5.006). Após essas exclusões, um total de 25.248 participantes foram incluídos na análise final, conforme mostrado na Figura 1.

**Figura 1:**
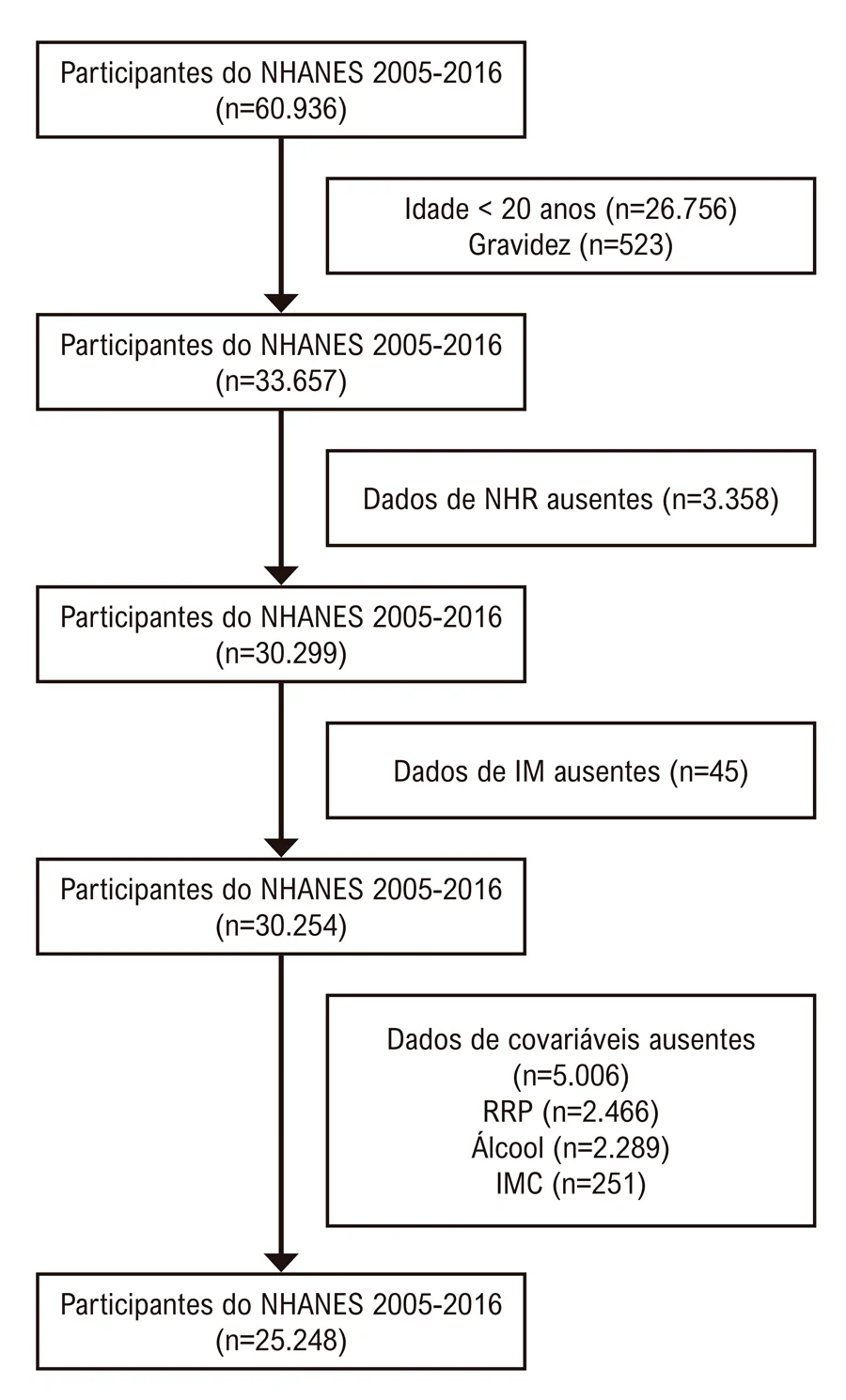
– Fluxograma da triagem de participantes. NHANES: National Health and Nutrition Examination Survey; RRP: razão de renda e pobreza; NHR: razão neutrófilo/colesterol HDL; IM: infarto do miocárdio; IMC: índice de massa corporal.

### NHR

A NHR é calculada usando a seguinte fórmula:


$$\mathrm{NHR}=\frac{\mathrm{Neu}(1000/\mu l)}{\text{ HDL - C }(\mathrm{mmol}/L)}$$

As contagens de neutrófilos e as concentrações de HDL-C foram obtidas por meio de testes laboratoriais padronizados, seguindo os protocolos do NHANES, garantindo precisão e reprodutibilidade.^22^

### IM

O histórico de IM foi determinado a partir das respostas dos participantes a um questionário sobre condições médicas. Os participantes que responderam “sim” à pergunta: “Algum médico ou outro profissional de saúde já lhe disse que você teve um ataque cardíaco (também chamado de infarto do miocárdio)?” foram considerados como tendo histórico de IM.^23^

### Covariáveis

Os potenciais fatores de confusão incluíram idade (modelada como uma variável contínua nas análises de regressão), sexo, raça/etnia e nível de escolaridade (abaixo do ensino médio, ensino médio ou superior),^24^ razão de renda familiar de pobreza (RRP) (<1,3, 1,3–3,49, ≥3,5),^25^ estado civil (casado ou solteiro)^24^ e fatores clínicos e de estilo de vida, incluindo hipertensão,^26^ diabetes,^27^ status de fumante^25^ consumo de álcool,^28^ atividade física^27^ e índice de massa corporal (IMC, contínuo). A hipertensão foi definida como uma resposta afirmativa a um diagnóstico médico, uso de medicamentos anti-hipertensivos ou pressão arterial medida ≥140/90 mmHg. O diabetes foi definido com base em autodiagnóstico, uso de insulina ou hipoglicemiantes orais ou critérios laboratoriais, incluindo glicemia de jejum ≥ 7,0 mmol/L ou HbA1c ≥ 6,5%. O consumo de álcool foi classificado como não bebedores (menos de 12 doses por ano) e bebedores (12 ou mais doses por ano). O tabagismo foi classificado como nunca fumou, fumante atual ou ex-fumante, com base nas respostas sobre o consumo de ≥100 cigarros ao longo da vida e o comportamento atual de fumar. A atividade física foi definida como a participação em atividades vigorosas durante o trabalho ou lazer que aumentassem substancialmente a respiração ou a frequência cardíaca por pelo menos 10 minutos consecutivos.

### Análise estatística

As análises foram conduzidas de acordo com o plano de amostragem complexo do NHANES, incorporando estratificação, agrupamento e ponderação da pesquisa para garantir a representatividade nacional. A normalidade das variáveis contínuas foi avaliada por meio de métodos gráficos e estatísticas descritivas sumárias. Devido ao grande tamanho da amostra, foram aplicados testes paramétricos, dada a sua robustez a desvios moderados da normalidade. As características basais foram resumidas por status de IM e por quartis de NHR (Q1–Q4), com variáveis categóricas apresentadas como contagens e porcentagens e variáveis contínuas como médias com desvios padrão. Para variáveis contínuas, foi utilizada a análise de variância (ANOVA) de uma via ponderada pela amostra, e os tamanhos de efeito gerais (ω^2^) foram calculados. Para comparações binárias por status de IM, foram realizados testes-t de amostras independentes ponderados pela amostra, com o d de Cohen calculado como tamanho de efeito. Para variáveis categóricas, foram realizados testes qui-quadrado ponderados pela amostra, com o V de Cramer calculado como tamanho de efeito. Os valores de p foram ajustados para comparações múltiplas utilizando o método da taxa de falsos positivos (TFP). Quando diferenças gerais foram identificadas, as comparações pareadas foram interpretadas descritivamente, sem testes post-hoc formais, devido à natureza exploratória da análise. Foram utilizados modelos de regressão logística ponderada para examinar as associações de NHR, neutrófilos e HDL-C com IM, levando em consideração o desenho amostral probabilístico complexo e multiestágios do NHANES por meio do uso de pesos de pesquisa recomendados. As covariáveis foram selecionadas a priori com base na relevância clínica e na literatura prévia, incluindo fatores demográficos (idade, sexo, raça/etnia, escolaridade e índice de pobreza familiar), fatores de estilo de vida (tabagismo, consumo de álcool e atividade física), estado civil, IMC contínuo e comorbidades (hipertensão e diabetes). Uma estratégia sequencial de ajuste multivariável foi aplicada: o Modelo 1 não foi ajustado; o Modelo 2 foi ajustado para sexo, idade, raça/etnia, escolaridade e índice de pobreza familiar; e o Modelo 3 foi ajustado adicionalmente para fatores de estilo de vida, IMC, estado civil e comorbidades. As potenciais associações não lineares entre NHR e IM foram avaliadas usando splines cúbicos restritos (SCR), com nós posicionados nos percentis 25, 50 e 75. A análise de limiar foi realizada utilizando uma abordagem de regressão segmentada para identificar o valor de NHR no qual a associação com IM diferia abaixo e acima do limiar. Foram realizadas análises de subgrupos definidos por idade (<60 anos vs. ≥60 anos), sexo, raça/etnia, tabagismo e atividade física. Testes de interação foram realizados para cada variável de subgrupo separadamente, e todas as análises de subgrupos foram consideradas exploratórias devido às múltiplas comparações. Além disso, análises de sensibilidade utilizando imputação múltipla para lidar com covariáveis faltantes foram conduzidas para avaliar a robustez dos principais achados. Todas as análises estatísticas foram realizadas utilizando o software R (versão 4.2.1). Um valor de P bicaudal < 0,05 foi considerado estatisticamente significativo.

## Resultados

### Características basais da população do estudo

Um total de 25.248 participantes foram incluídos, dos quais 1.073 tinham histórico de IM. Comparados aos participantes sem IM, aqueles com IM eram mais velhos e apresentavam menores índices de pobreza. Eles também apresentavam IMC mais elevado. e NHR mais elevada. Diferenças significativas também foram observadas em raça, escolaridade, sexo, tabagismo, atividade física, diabetes e hipertensão, enquanto o consumo de álcool e o estado civil apresentaram variação mínima. Quando os participantes foram estratificados por quartis de NHR, o IMC aumentou progressivamente, enquanto a idade e a RRP diminuíram progressivamente ao longo dos quartis. A prevalência de IM, sexo masculino, diabetes, tabagismo e hipertensão aumentou progressivamente nos quartis mais altos de NHR, enquanto as distribuições de raça e escolaridade sofreram alterações moderadas. O consumo de álcool e o estado civil variaram minimamente entre os quartis. Coletivamente, uma NHR mais elevada foi associada à predominância masculina, perfis cardiometabólicos adversos, padrões de estilo de vida não saudáveis e aumento da prevalência de IM, o que reflete tanto diferenças transversais por status de IM quanto associações graduais entre os quartis de NHR (Tabelas 1 - 2).

**Tabela 1:** – Características dos participantes de acordo com o infarto do miocárdio

Características	IM (Sim) n=1073	IM (Não) n=24175	Valor de p	Tamanho do efeito
**Idade, (anos)**	66,84 (12,27)	48,84 (17,51)	<0,001	-1.190
**RRP**	2,12 (1,50)	2,56 (1,63)	<0,001	0.278
**IMC,(kg/m**^**2**^**)**	30,23 (7,06)	29,12 (6,90)	<0,001	-0,159
**NHR**	4.04(2.16)	3,44(2.16)	<0,001	-0,275
**Raça, n(%)**			<0,001	0,064
Mexicano-americano	98 ( 9.10)	3825 (15,80)		
Outros hispânicos	83 ( 7,70)	2234 ( 9.20)		
Branco não hispânico	641 (59,70)	10949 (45,30)		
Negro não hispânico	198 (18,50)	4915 (20,30)		
Outra raça	53 ( 4,90)	2252 ( 9,30)		
**Educação, n(%)**			<0,001	0,052
Abaixo do ensino médio	375 (34,90)	5763 (23,80)		
Ensino médio completo ou superior	698 (65,10)	18412 (76,20)		
**Gênero, n(%)**			<0,001	0,069
Masc.	713 (66,40)	11935 (49,40)		
Fem.	360 (33,60)	12240 (50,60)		
**Tabagismo n(%)**			<0,001	0,099
Fumante	262 (24,40)	5064 (20,90)		
Ex-fumante	462 (43,10)	5809 (24,00)		
Não-fumante	349 (32,50)	13302 (55,00)		
**Atividade física, n(%)**			<0,001	0,071
Alta	361 (33,60)	12395 (51,30)		
Baixa	712 (66,40)	11780 (48,70)		
**Diabetes, n(%)**			<0,001	0,134
Sim	443 (41,30)	3897 (16.10)		
Não	630 (58,70)	20278 (83,90)		
**Bebida, n(%)**			0.715	0.003
Sim	778 (72,50)	17393 (71,90)		
Não	295 (27,50)	6782 (28.10)		
**Status conjugal, n(%)**			0.003	0.019
Sim	597 (55,60)	14548 (60,20)		
Não	476 (44,40)	9627 (39,80)		
**Hipertensão, n(%)**			<0,001	0,152
Sim	841 (78,40)	9915 (41.00)		
Não	232 (21,60)	14260 (59.00)		

IM: infarto do miocárdio; NHR: relação neutrófilo/colesterol HDL; HDL-C: colesterol da lipoproteína de alta densidade; RRP: índice de pobreza; IMC: índice de massa corporal; OR: odds ratio; IC: intervalo de confiança

**Tabela 2:** – Características dos participantes de acordo com os quartis do NHR

Características	NHR				Valor de p	Tamanho do efeito
	Q1(n=6312)	Q2(n=6312)	Q3(n=6312)	Q4(n=6312)		
**Idade, média (DP)**	50,74 (17,68)	50,21 (17,89)	49,36 (17,87)	48,12 (17,24)	<0,001	0,003
**RRP, média (DP)**	2,77 (1,66)	2,64 (1,64)	2,47 (1,61)	2,27 (1,56)	<0,001	0,013
**IMC (kg/m**^**2**^**), média (DP)**	26,70 (5,87)	28,41 (6,31)	29,71 (6,61)	31,85 (7,67)	<0,001	0,074
**IM, n(%)**					<0,001	0,061
Sim	170 (2,70)	247 (3,90)	271 (4,30)	385 (6.10)		
Não	6142 (97,30)	6065 (96,10)	6041 (95,70)	5927 (93,90)		
**Gênero, n(%)**					<0,001	0,159
Masc.	2458 (38,90)	2995 (47,40)	3368 (53,40)	3827 (60,60)		
Fem.	3854 (61,10)	3317 (52,60)	2944 (46,60)	2485 (39,40)		
**Raça, n(%)**					<0,001	0,133
Mexicano-americano	632 (10,00)	904 (14,30)	1199 (19,00)	1188 (18,80)		
Negro não hispânico	2148 (34,00)	1308 (20,70)	947 (15,00)	710 (11,20)		
Branco não hispânico	2450 (38,80)	2870 (45,50)	2983 (47,30)	3287 (52,10)		
Outros hispânicos	448 (7.10)	602 (9,50)	649 (10,30)	618 (9,80)		
Outra raça	634 (10,00)	628 (9,90)	534 (8,5)0)	509 (8.10)		
**Educação, n(%)**					<0,001	0,071
Abaixo do ensino médio	1273 (20,20)	1464 (23,20)	1604 (25,40)	1797 (28,50)		
Ensino médio completo ou superior	5039 (79,80)	4848 (76,80)	4708 (74,60)	4515 (71,50)		
**Tabagismo, n(%)**					<0,001	0,120
Fumante	904 (14,30)	1086 (17,20)	1337 (21,20)	1999 (31,70)		
Ex-fumante	1525 (24,20)	1569 (24,90)	1637 (25,90)	1540 (24,40)		
Não-fumante	3883 (61,50)	3657 (57,90)	3338 (52,90)	2773 (43,90)		
**Atividade física, n(%)**					<0,001	0,103
Alta	3588 (56,80)	3340 (52,90)	3126 (49,50)	2702 (42,80)		
Baixa	2724 (43,20)	2972 (47,10)	3186 (50,50)	3610 (57,20)		
**Diabetes, n(%)**					<0,001	0,137
Sim	667 (10,60)	947 (15,00)	1170 (18,50)	1556 (24,70)		
Não	5645 (89,40)	5365 (85,00)	5142 (81,50)	4756 (75,30)		
**Bebida, n(%)**					<0,001	0,025
Sim	4448 (70,5)	4525 (71,7)	4550 (72,1)	4648 (73,6)		
Não	1864 (29,5)	1787 (28.3)	1762 (27,9)	1664 (26,4)		
**Status conjugal, n(%)**					<0,001	0,041
Sim	3583 (56,80)	3794 (60,10)	3849 (61,00)	3919 (62,10)		
Não	2729 (43,20)	2518 (39,90)	2463 (39,00)	2393 (37,90)		
**Hipertensão, n(%)**					<0,001	0,055
Sim	2482 (39,30)	2625 (41,60)	2692 (42,60)	2957 (46,80)		
Não	3830 (60,70)	3687 (58,40)	3620 (57,40)	3355 (53,20)		

IM: infarto do miocárdio; NHR: relação neutrófilo/colesterol HDL; HDL-C: colesterol da lipoproteína de alta densidade; RRP: índice de pobreza; IMC: índice de massa corporal; OR: odds ratio; IC: intervalo de confiança

### Associação entre NHR, Neutrófilos, HDL-C, e IM

A NHR mais elevada foi consistentemente associada a maiores chances de IM em modelos não ajustados e parcialmente ajustados (Modelos 1 e 2). Após o ajuste completo para covariáveis demográficas e clínicas (Modelo 3), os participantes no quartil mais alto de NHR apresentaram 49% mais chances de IM em comparação com aqueles no quartil mais baixo, com uma tendência significativa de dose-resposta entre os quartis. Para a contagem de neutrófilos, a associação com o IM foi atenuada no modelo totalmente ajustado. Os participantes no quartil mais alto apresentaram uma probabilidade 11% maior de IM, embora não significativa, em comparação com o quartil mais baixo. A tendência entre os quartis não foi estatisticamente significativa. Para o HDL-C, níveis mais elevados foram consistentemente associados a menores chances de IM. No modelo totalmente ajustado, os participantes no quartil mais alto apresentaram 38% menos chances de IM em comparação com o quartil mais baixo. e apresentaram uma tendência significativa de dose-resposta entre os quartis. Esses resultados indicam que a NHR apresentou associações mais consistentes com o IM do que a contagem de neutrófilos ou o HDL-C isoladamente (Tabela 3).

**Tabela 3:** – Análise de regressão logística de NHR, neutrófilos e HDL-C com IM

	Modelo 1	Modelo 2	Modelo 3
**NHR Interquartil**	**OR(IC 95%) Valor de p**	**OR(IC 95%) Valor de p**	**OR(IC 95%) Valor de p**
Q1 <2,139	Ref	Ref	Ref
Q2 2139~3,067	1.52(1.18, 1.96) 0,001	1.46(1.11, 1.920 008	1.26(0,95, 1.67) 0,105
Q3 3,067~4.308	1.70(1,35, 2.14) <0,001	1.64(1.29, 2.08) <0,001	1.27(0,99, 1.61) 0,052
Q4 >4.308	2.38(1,85, 3.06) <0,001	2.39(1,82, 3.14) <0,001	1.49(1.11,2,00) 0,008
P para tendência	<0,001	<0,001	0,007
**Neutrófilos Interquartil**			
Q1 <3.1	Ref	Ref	Ref
Q2 3.1~4.0	1,13 (0,87, 1,46) 0,353	1,05 (0,81, 1,36) 0,706	0,91 (0,70, 1,19) 0,497
Q3 4.0~5.1	1,52 (1,20, 1,94) <0,001	1,34 (1,04, 1,72) 0,025	1,07 (0,83, 1,38) 0,602
Q4 >5.1	1,74 (1,39, 2,19) <0,001	1,66 (1,31, 2,09) <0,001	1,11 (0,87, 1,43) 0,397
P para tendência	<0,001	<0,001	0,178
**HDL-C Interquartil**			
Q1 <1.06	Ref	Ref	Ref
Q2 1.06~1,29	0,75 (0,60, 0,93) 0,010	0,71 (0,56, 0,90) 0,005	0,80 (0,63, 1,01) 0,060
Q3 1,29~1,60	0,49 (0,40, 0,61) <0,001	0,49 (0,39, 0,62) <0,001	0,61 (0,48, 0,77) <0,001
Q4 >1,60	0,49 (0,38, 0,63) <0,001	0,45 (0,35, 0,59) <0,001	0,62 (0,47, 0,81) <0,001
P para tendência	<0,001	<0,001	<0,001

O Modelo 1 não foi ajustado. Modelo 2 ajustado por sexo, idade, raça, índice de pobreza/renda familiar e nível de escolaridade. Modelo 3 ajustado para todas as covariáveis. IM: infarto do miocárdio; NHR: relação neutrófilo/colesterol HDL; HDL-C: colesterol da lipoproteína de alta densidade; IMC: índice de massa corporal; OR: odds ratio; IC: intervalo de confiança.

A análise SCR também foi realizada para avaliar a possível associação não linear entre NHR e IM. Como mostrado na Figura 2, após o ajuste para todas as covariáveis, tanto a associação geral quanto o componente não linear foram estatisticamente significativos, sugerindo uma relação não linear modesta entre NHR e IM. A análise de limiar identificou um ponto de inflexão baseado nos dados em uma NHR de 6,93. Abaixo desse ponto, uma NHR mais alta foi associada a maiores chances de IM, enquanto a associação acima do ponto de inflexão não foi estatisticamente significativa. No entanto, essa descoberta de limiar deve ser interpretada com cautela, como exploratória e descritiva, e não como evidência de um ponto de corte clinicamente significativo, particularmente devido ao número relativamente limitado de participantes na cauda superior da distribuição da NHR. Como mostrado na Tabela 4, o teste da razão de verossimilhança corroborou o modelo de limiar.

**Figura 2:**
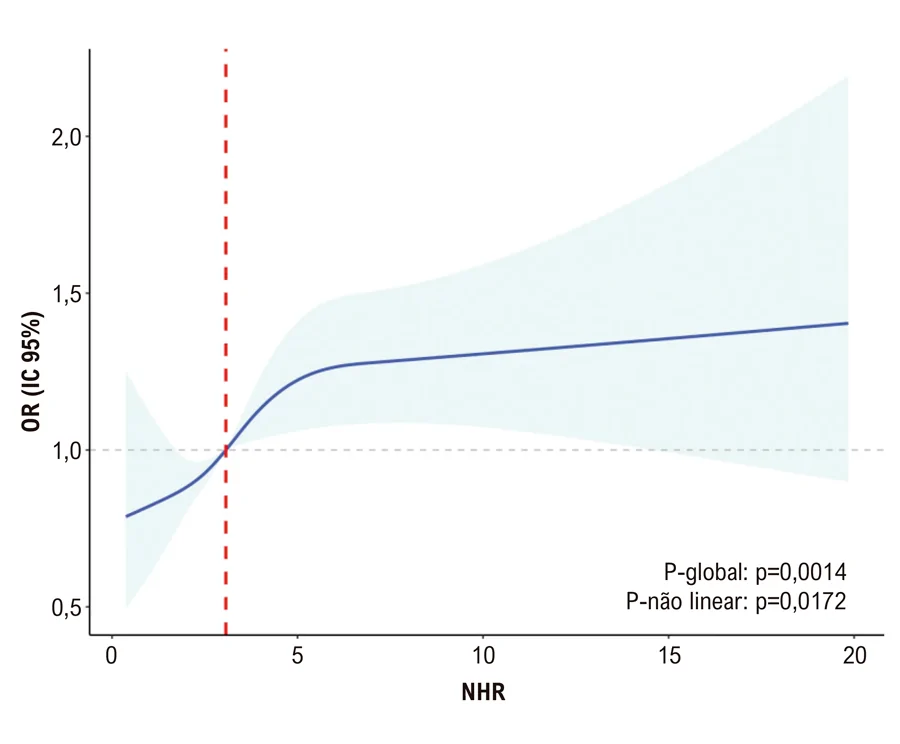
– Curvas SCR ilustrando a associação ajustada entre a NHR e a probabilidade de infarto do miocárdio na coorte completa (n = 25.248), com três nós. A linha contínua representa a razão de chances (RC) e as linhas tracejadas indicam os intervalos de confiança de 95%. HNR: razão neutrófilo-colesterol da lipoproteína de alta densidade; IM: infarto do miocárdio; RC: razão de chances; IC: intervalo de confiança.

**Tabela 4:** – Análise de limiar de NHR e chances de infarto do miocárdio

	OR(IC 95%)	Valor de p
Ponto de inflexão	6,93	
NHR < 6,93	1,10 (1,05, 1,15)	<0,001
NHR ≥ 6,93	0,99 (0,92, 1,03)	0,717
Razão de log-verossimilhança		<0,001

### Análise de subgrupos

Análises adicionais de subgrupos sugeriram que a associação entre NHR e IM pode variar entre os subgrupos. Como mostrado na Figura 3, associações positivas entre NHR e IM foram observadas em diversos subgrupos, incluindo participantes com idade ≥ 60 anos, mulheres, indivíduos com IMC > 25 kg/m^2^, indivíduos com baixa atividade física, indivíduos com hipertensão, indivíduos com diabetes e indivíduos sem histórico de acidente vascular cerebral (AVC). No entanto, dada a natureza exploratória dessas análises e os múltiplos testes de interação realizados, os resultados dos subgrupos devem ser interpretados com cautela. Entre os subgrupos examinados, a interação por sexo foi estatisticamente significativa, sugerindo que a associação pode ser mais forte em mulheres.

**Figura 3:**
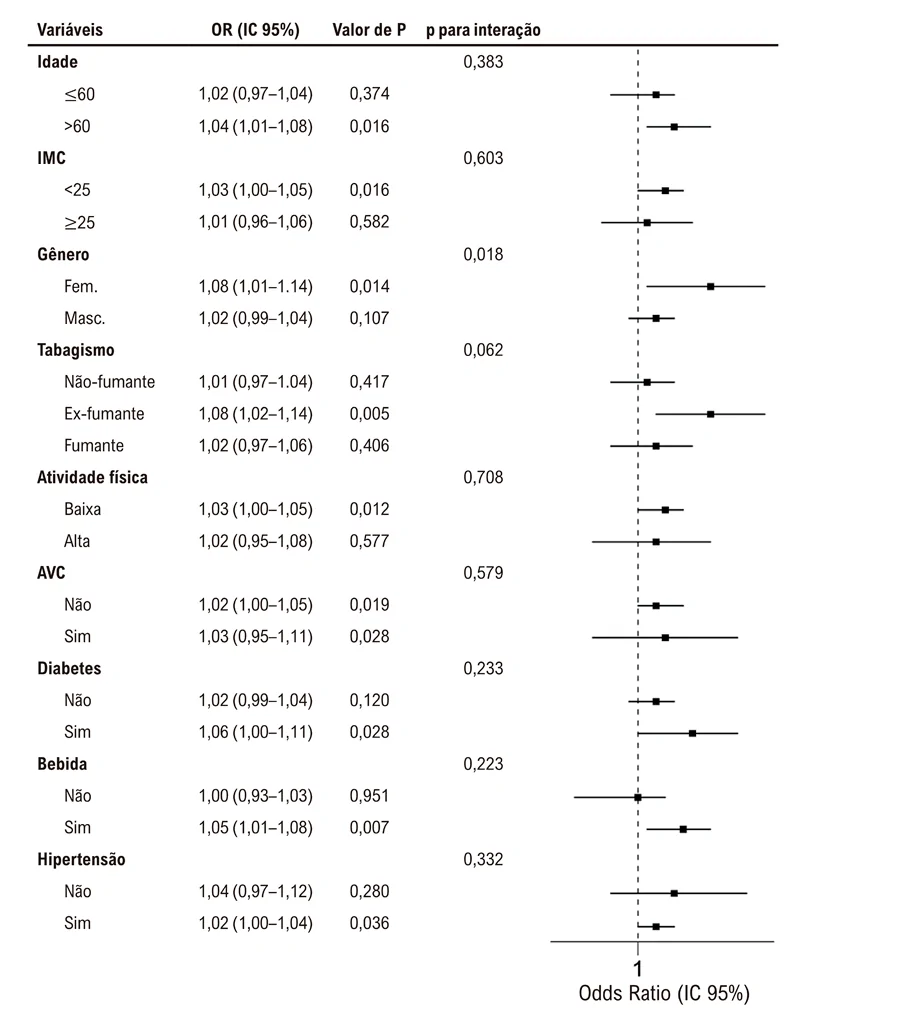
– Análise de subgrupos da associação entre NHR e IM. Razões de chances Os intervalos de confiança de 95% são apresentados para cada subgrupo predefinido. Os valores de P específicos para cada componente indicam a significância estatística da associação entre NHR e IM em cada subgrupo, enquanto os valores de P para interação avaliam a possível modificação do efeito entre os subgrupos. NHR: razão neutrófilo-colesterol da lipoproteína de alta densidade; IM: infarto do miocárdio; OR: odds ratio; IC: intervalo de confiança.

### Análise de sensibilidade

Como parte das análises de sensibilidade, utilizamos imputação múltipla por equações encadeadas para imputar covariáveis faltantes, mantendo os valores observados de NHR e IM e incorporando o desenho da pesquisa NHANES e os pesos de amostragem em cada conjunto de dados imputados. Os resultados foram substancialmente consistentes com as descobertas da análise primária de casos completos (Tabela Suplementar 2). No modelo totalmente ajustado, uma NHR mais elevado permaneceu associada a maiores chances de IM. Os participantes no quartil mais alto apresentaram chances significativamente maiores de IM do que aqueles no quartil mais baixo (OR 1,536, IC 95% 1,141–2,067; p=0,005), e a tendência entre os quartis também foi estatisticamente significativa (p=0,004). Em contraste, a contagem de neutrófilos isoladamente não foi significativamente associada ao IM após o ajuste completo, enquanto níveis mais elevados de HDL-C permaneceram inversamente associados ao IM. Essas descobertas corroboram a robustez dos achados primários.

Uma visão geral esquemática do projeto experimental e das principais conclusões é apresentada na Figura Central.

## Discussão

Nesta análise transversal de 25.248 participantes do NHANES, níveis mais elevados de NHR foram associados a maiores chances de IM, refletindo, portanto, uma doença prevalente em vez de incidente. Após ajuste multivariável, os participantes no quartil mais alto de NHR apresentaram chances significativamente maiores de IM do que aqueles no quartil mais baixo, enquanto a contagem de neutrófilos isoladamente não apresentou associação significativa com IM, e os níveis de HDL-C apresentaram associação inversa com IM. A análise de spline cúbica restrita sugeriu uma associação não linear modesta entre NHR e IM, com um ponto de inflexão determinado pelos dados em uma NHR de aproximadamente 6,93. Como a maioria dos participantes apresentou valores de NHR abaixo desse ponto e relativamente poucos estavam na cauda superior, a não linearidade observada deve ser interpretada com cautela, como exploratória e descritiva, e não como evidência de um ponto de corte clinicamente significativo. Análises de subgrupos sugeriram que a associação pode ser mais forte em mulheres; no entanto, esses achados devem ser interpretados como exploratórios. As análises de sensibilidade também produziram resultados substancialmente consistentes, corroborando a robustez dos achados principais. De forma geral, nossos resultados corroboram a associação entre uma NHR mais elevada e maiores chances de IM nessa população.

Nossos resultados corroboram e ampliam as evidências existentes sobre a relevância clínica da NHR. Dados de coorte baseados na comunidade de Taiwan indicam que a NHR supera a razão neutrófilo-linfócito (NLR) na predição do risco de doença cardiovascular.^29^ Estudos clínicos em pacientes com doença arterial coronariana (DAC) associaram uma NHR mais elevada a uma estenose coronariana mais grave, conforme refletido pelos escores de Gensini (AUC~0,73).^30^ Em pacientes com diabetes tipo 2 e síndrome coronariana aguda, a NHR também demonstrou forte valor preditivo e diagnóstico.^13^ Da mesma forma, um estudo de coorte chinês descobriu que a NHR elevada previa desfechos cardiovasculares adversos em indivíduos pré-diabéticos.^31^ Mais recentemente, dados do NHANES revelaram que níveis elevados de NHR, combinados com comprometimento cognitivo, estavam associados a uma mortalidade por todas as causas substancialmente maior em idosos.^32^ Ao contrário de estudos anteriores, conduzidos principalmente em populações clínicas ou de alto risco, nosso estudo utiliza uma amostra representativa nacional e identifica uma forte associação entre a NHR e o IM na população adulta geral dos EUA, destacando sua potencial relevância epidemiológica. Marcadores tradicionais, como LDL-C e PCR-as, refletem a carga lipídica e a inflamação sistêmica separadamente, enquanto a NHR integra a atividade inflamatória inata e uma fração lipídica antiaterogênica em um único índice, capturando o equilíbrio entre as vias pró-inflamatórias e protetoras e potencialmente explicando sua relevância em estudos populacionais.

Evidências crescentes sugerem que a NHR pode estar associada ao IM por meio de mecanismos que envolvem inflamação, metabolismo lipídico desregulado e disfunção endotelial vascular.^33,34^

Os neutrófilos estão entre as primeiras células do sistema imune a infiltrar o miocárdio infartado e atingem níveis máximos nas 24 horas seguintes ao IM. Ao infiltrar a área do infarto, os neutrófilos liberam complexos de calprotectina (S100A8/A9), que ativam o receptor do tipo Toll 4 (TLR4) e os inflamassomos do tipo NOD (NLRs), promovendo, assim, a secreção de citocinas pró-inflamatórias, como a interleucina-1β. Essas citocinas estimulam a hematopoiese na medula óssea, sustentando a produção de neutrófilos e amplificando as respostas inflamatórias, que, por sua vez, exacerbam a lesão miocárdica.^35,36^ Neutrófilos ativados também liberam proteases e mediadores pró-inflamatórios que polarizam os macrófagos em direção a um fenótipo pró-inflamatório (tipo M1), inibindo assim o reparo tecidual.^37^ Estudos relataram que, no IM com supradesnivelamento do segmento ST, neutrófilos e outras células inflamatórias aderem e transmigram através das células endoteliais por meio de integrinas. Durante esse processo, liberam citocinas pró-inflamatórias que comprometem a função endotelial, exacerbam a inflamação e a lesão miocárdica, agravam a lesão de isquemia-reperfusão e promovem a remodelação ventricular.^4^

Por outro lado, o HDL-C exerce efeitos cardioprotetores através de suas moléculas bioativas, ativando vias de sinalização celular e inibindo a abertura do poro de transição da permeabilidade mitocondrial, o que, por sua vez, reduz a apoptose e a necrose dos cardiomiócitos. Além disso, o HDL-C suprime a ativação do NF-κB, reduzindo assim a liberação de citocinas inflamatórias e atenuando a inflamação miocárdica.^38^ O HDL-C também facilita o transporte reverso de colesterol, removendo o colesterol das placas arteriais e, assim, exercendo efeitos protetores contra o IM.^39^

Análises de subgrupos sugeriram que a associação entre NHR e IM pode ser mais forte em mulheres do que em homens; no entanto, esses achados devem ser interpretados com cautela, pois as análises de subgrupos foram exploratórias e envolveram múltiplas comparações e testes de interação. Evidências anteriores indicam que mulheres com IM podem apresentar características de placa distintas das dos homens, com maior propensão à erosão da placa do que à ruptura. Aproximadamente 30% das mulheres com IM apresentam doença arterial coronariana não obstrutiva na angiografia, uma proporção maior do que a observada em homens, o que pode estar relacionado à disfunção microvascular ou ao espasmo da artéria coronariana.^40^ Além disso, acredita-se que o estrogênio confira proteção relativa contra doenças cardiovasculares em mulheres na pré-menopausa; essa proteção diminui após a menopausa, com o risco cardiovascular se aproximando do dos homens.^41^ Nos participantes com AVC, a associação entre NHR e IM não foi estatisticamente significativa; no entanto, esse achado não deve ser superinterpretado. Estudos anteriores sugerem que o AVC isquêmico agudo pode desencadear respostas imunológicas e relacionadas ao estresse que aumentam os níveis de neutrófilos circulantes.^42^ Além disso, a ativação neuroimune associada ao AVC pode alterar os perfis inflamatórios periféricos e, assim, afetar a especificidade da NHR nesse subgrupo.^43^ De modo geral, esses resultados de subgrupos devem ser considerados como geradores de hipóteses, e não como evidências de modificação definitiva do efeito biológico.

A robustez dos nossos resultados foi reforçada pela grande amostra nacionalmente representativa do NHANES e pelo ajuste para uma ampla gama de potenciais fatores de confusão. Este estudo sugere que a NHR pode servir como um biomarcador associado ao IM, com análises de subgrupos indicando que a associação pode ser mais forte em mulheres. No entanto, o delineamento transversal e a dependência do histórico de IM autorrelatado impedem a inferência causal, limitam a avaliação da temporalidade e impedem a diferenciação entre eventos de IM recentes e remotos. Além disso, como a NHR foi medida no momento do exame do NHANES, e não antes da ocorrência do IM, ela pode ter sido influenciada pelo próprio IM prévio, bem como por tratamentos subsequentes e mudanças comportamentais após o IM, incluindo terapia hipolipemiante e modificação do estilo de vida. Portanto, a causalidade reversa não pode ser excluída. O viés de seleção também pode ter resultado da exclusão de participantes com dados faltantes, e a presença de fatores de confusão residuais não pode ser totalmente descartada. Em particular, não conseguimos ajustar de forma consistente para a terapia hipolipemiante e para biomarcadores lipídicos e inflamatórios estabelecidos, como LDL-C, triglicerídeos e PCR-as, em todos os ciclos do NHANES incluídos. Esses fatores podem ter influenciado tanto o status de NHR quanto o de IM e, portanto, permanecem como potenciais fontes de confusão residual. Embora as análises do valor E tenham sugerido que uma confusão não mensurada relativamente forte seria necessária para explicar completamente a associação observada, algumas variáveis ajustadas podem estar na via causal, aumentando a possibilidade de sobreajuste. Por fim, o desempenho preditivo não foi avaliado formalmente e a generalização desses achados para além da população adulta dos EUA pode ser limitada.

## Conclusão

Em suma, nossos achados indicam que níveis mais elevados da razão neutrófilos/colesterol HDL estão associados a uma maior probabilidade de IM, observando-se um padrão não linear moderado ao longo da faixa de valores dessa razão. Tais associações persistiram após o ajuste multivariável e refletem a prevalência de IM, e não o risco de eventos incidentes. Análises de subgrupos sugeriram que a associação pode ser mais forte em mulheres; no entanto, esses resultados devem ser interpretados como exploratórios. Dado o delineamento transversal, não é possível inferir causalidade, sendo necessários estudos prospectivos para esclarecer as relações temporais e elucidar melhor os mecanismos subjacentes.

## * Material suplementar

Supplementary material 1 Para Tabela Suplementar 1, por favor, clique aqui.https://abccardiol.org/supplementary-material/2026/12308/2025-0718_AO_Supplementary_Table1.pdf

Material suplementar Para Tabela Suplementar 2, por favor, clique aqui. https://abccardiol.org/supplementary-material/2026/12308/2025-0718_AO_Supplementary_Table2.pdf
