# Supplementary material for: Associação entre a Relação Neutrófilo-Colesterol HDL e Infarto do Miocárdio: Um Estudo Transversal
Source: Arq Bras Cardiol. 2026 Aug 24;123(8):e20250718. [Article in Portuguese] doi: 10.36660/abc.20250718 (PMC13671726; doi:10.36660/abc.20250718)
Supplement: Supplementary file 3 [file 0066-782x-abc-123-8-e20250718-suppl02-en.pdf]

Supplementary Table 2 Logistic regression analyses of NHR, neutrophils, and HDL-C with MI (multiple imputation for missing covariates)

	**Model1**	**Model2**	**Model3**
**NHR**	OR (95% CI)	OR (95% CI)	OR (95% CI)
Inter quartile	P-value	P-value	P-value
Q1<br>< 2.136	Ref	Ref	Ref
Q2	1.461 (1.153, 1.851)	1.391 (1.080, 1.792)	1.219 (0.942, 1.576)
2.136~3.066	0.002	0.011	0.132
Q3	1.655 (1.300, 2.107)	1.576 (1.223, 2.030)	1.232 (0.953, 1.594)
3.066~4.310	<0.001	<0.001	0.112
Q4	2.420 (1.878, 3.118)	2.434 (1.843, 3.215)	1.536 (1.141, 2.067)
>4.310	<0.001	<0.001	0.005
P for trend	<0.001	<0.001	0.004
**Neutrophils**			
Inter quartile			
Q1<br>< 3.100	Ref	Ref	Ref
Q2	1.278 (0.982, 1.662)	1.172 (0.899, 1.528)	1.028 (0.786, 1.345)
3.100~4.000	0.068	0.242	0.842
Q3	1.496 (1.223, 1.832)	1.310 (1.069, 1.607)	1.067 (0.864, 1.318)
4.000~5.100	<0.001	0.009	0.549
Q4	1.827 (1.471, 2.268)	1.732 (1.386, 2.166)	1.172 (0.922, 1.489)
>5.100	<0.001	<0.001	0.195
P for trend	<0.001	<0.001	0.148
**HDL-C**			
Inter quartile			
Q1<br>< 1.060	Ref	Ref	Ref
Q2	0.712 (0.579, 0.875)	0.684 (0.553, 0.847)	0.774 (0.624, 0.960)
1.060~1.290	0.001	<0.001	0.020
Q3	0.498 (0.406, 0.612)	0.493 (0.395, 0.617)	0.629 (0.505, 0.783)
1.290~1.600	<0.001	<0.001	<0.001
Q4	0.461 (0.361, 0.589)	0.427 (0.330, 0.554)	0.591 (0.448, 0.780)
>1.600	<0.001	<0.001	<0.001
P for trend	<0.001	<0.001	<0.001

Model 1 was unadjusted.

Model 2 adjusted for sex, age, race, family poverty income ratio, and education level.

Model 3 adjusted for all covariates.
