# Supplementary material for: Associação entre a Relação Neutrófilo-Colesterol HDL e Infarto do Miocárdio: Um Estudo Transversal
Source: Arq Bras Cardiol. 2026 Aug 24;123(8):e20250718. [Article in Portuguese] doi: 10.36660/abc.20250718 (PMC13671726; doi:10.36660/abc.20250718)
Supplement: Translation

# Translation: Association between the Neutrophil-to-HDL-Cholesterol Ratio and Myocardial Infarction: A Cross-Sectional Study

**DOI:** 10.36660/abc.20250718i

**Authors:** Youpeng Wang, Qian Yin, Ying Lei, Juncang Duan

**Affiliations:** 1 China Department of Cardiology – Second District – Affiliated Jinhua Hospital, Zhejiang University School of Medicine, Jinhua – China

**Keywords:** Myocardial Infarction, Neutrophils, HDL Cholesterol, Nutrition Surveys

## Abstract

**Background:**

Myocardial infarction (MI) remains a major global health burden. Inflammation and lipid metabolism’s roles in MI pathogenesis have drawn more attention. The neutrophil-to-high-density lipoprotein cholesterol ratio (NHR) is a new composite biomarker integrating inflammation and lipid metabolism markers, but its association with MI is unclear.

**Objectives:**

We investigated the association between NHR and MI.

**Methods:**

We analyzed data from 25,248 eligible participants from NHANES between 2005 and 2016. A history of MI was determined using questionnaire data, and NHR values were derived from laboratory measurements. Logistic regression models were used to assess the associations of NHR, neutrophil count, and high-density lipoprotein cholesterol (HDL-C) with the odds of MI. Restricted cubic spline (RCS) analysis was used to evaluate potential nonlinear associations. Subgroup analyses and interaction tests were performed to explore potential heterogeneity across demographic and clinical subgroups. Sensitivity analyses were performed to evaluate the robustness of the primary findings. A two-sided P value of <0.05 was considered statistically significant.

**Results:**

Higher NHR was independently associated with higher odds of MI. In the fully adjusted model, participants in the highest NHR quartile had 49% higher odds of MI than those in the lowest quartile. Neutrophil count alone was not significantly associated with MI, whereas HDL-C levels were inversely associated with MI. RCS suggested a modest nonlinear association between NHR and MI. Although a data-driven inflection point was identified at 6.93, this result should be interpreted as exploratory and descriptive rather than a clinically meaningful threshold. Subgroup analyses suggested a stronger association in women, and the interaction by sex was statistically significant. Sensitivity analyses confirmed the robustness of these findings.

**Conclusion:**

In this large, representative sample, elevated NHR was independently associated with higher odds of MI, suggesting that NHR may serve as a potentially informative epidemiological marker for MI. Prospective studies are needed to determine causality and elucidate the underlying mechanisms.

## Introduction

Myocardial infarction (MI) remains one of the leading causes of cardiovascular morbidity and mortality, imposing a substantial burden on global public health.^1^ According to the Global Burden of Disease Study, ischemic heart disease caused 9.14 million deaths worldwide in 2019, accounting for 49.2% of all cardiovascular-related deaths, and the burden of ischemic heart disease continues to rise.^2^ MI places considerable strain on healthcare systems and families and contributes substantially to premature mortality.^3^ Therefore, identifying factors associated with MI is essential for informing effective early prevention strategies.

Recent studies have demonstrated that inflammatory cells play a key role in MI progression. Excessive infiltration of neutrophils and lymphocytes, together with abnormal activation of the complement system, markedly exacerbates myocardial injury. Moreover, these cells and their secreted cytokines and chemokines collectively regulate myocardial repair and remodeling, and dysregulation of these processes is a major driver of adverse ventricular remodeling and heart failure.^4,5^ Lipid metabolism is also closely associated with MI. Specifically, long-chain n-3 polyunsaturated fatty acids and stearic acid are inversely associated with MI risk, whereas arachidonic acid is positively correlated.^6^ Moreover, elevated levels of very low-density lipoproteins, low-density lipoproteins and their cholesterol content, as well as triglycerides, are associated with an increased risk of MI, whereas large-particle high-density lipoprotein cholesterol (HDL-C) is inversely associated with MI risk.^7^

Both inflammation and dyslipidemia are key contributors to the pathogenesis of MI.^8^ The neutrophil-to–high-density lipoprotein cholesterol ratio (NHR) is a novel composite biomarker that integrates these two pathways and may reflect synergistic interactions that are not captured by individual biomarkers. Neutrophils infiltrate the infarcted myocardium and release pro-inflammatory cytokines and proteases that exacerbate myocardial injury. In contrast, HDL-C exerts cardiovascular protective effects by inhibiting nuclear factor kappa-B (NF-κB) activation, modulating inflammatory cytokine responses, and promoting reverse cholesterol transport.^9-12^ By integrating these two pathways, NHR may reflect the net balance between pro-inflammatory and protective mechanisms involved in MI. Previous studies have demonstrated the utility of NHR in assessing metabolic abnormalities in type 2 diabetes,^13^ predicting atherosclerotic plaque stability,^14^ and evaluating sepsis prognosis.^15^ Moreover, NHR has been associated with adverse cardiovascular outcomes, including coronary artery disease and plaque vulnerability in atherosclerosis.^16-19^ Although NHR has not yet been widely adopted in routine clinical practice, its derivation from standard laboratory measurements makes it a convenient and potentially practical biomarker for cardiovascular risk assessment.

However, the relationship between NHR and MI has not been fully elucidated in large, nationally representative populations. Although NHR has been investigated in other cardiovascular and metabolic contexts, its specific association with myocardial infarction remains incompletely characterized. To address this gap, we analyzed data from the National Health and Nutrition Examination Survey (NHANES) to assess whether higher NHR is associated with increased odds of MI among U.S. adults. We hypothesized that elevated NHR is associated with higher odds of myocardial infarction after adjustment for established cardiovascular risk factors, and that these associations may vary by sex and age. These analyses are intended to examine the epidemiological relevance of NHR and to generate hypotheses for future prospective and mechanistic studies.

## Materials and Methods

### Data source and study population

NHANES is a nationally representative health surveillance program conducted by the National Center for Health Statistics (NCHS) under the auspices of the U.S. Centers for Disease Control and Prevention (CDC).^20^ The survey integrates structured interviews, physical examinations, and laboratory tests to collect comprehensive health and nutritional data from the non-institutionalized civilian population of the United States, thereby enabling monitoring of national health status, nutritional levels, and disease prevalence trends. All survey instruments and protocols are standardized and publicly documented by the NCHS. This cross-sectional study was reported in accordance with the Strengthening the Reporting of Observational Studies in Epidemiology (STROBE) guidelines (Supplementary Table 1).^21^

In this study, we utilized data from NHANES cycles between 2005 and 2016, which initially included 60,936 participants. We excluded individuals who were under 20 years of age (n = 26,756), pregnant (n = 523), missing NHR data (n = 3,358), missing MI data (n = 45), and those with missing covariate information (n = 5,006). After these exclusions, a total of 25,248 participants were included in the final analysis, as shown in Figure 1.

**Figure 1:** – Flow diagram of participant screening. NHANES: National Health and Nutrition Examination Survey; PIR: poverty income ratio; NHR: neutrophil-to-hdl-cholesterol ratio; MI: myocardial infarction; BMI: body mass index.

### NHR

NHR is calculated using the following formula:


$$\mathrm{NHR}=\frac{\mathrm{Neu}(1000/\mu l)}{\text{ HDL - C }(\mathrm{mmol}/L)}$$

Both neutrophil counts and HDL-C concentrations were obtained through standardized laboratory testing following NHANES protocols, ensuring accuracy and reproducibility.^22^

### MI

MI status was ascertained from participants’ responses to a medical conditions questionnaire. Participants who answered “yes” to the question, “Has a doctor or other health professional ever told you that you had a heart attack (also called myocardial infarction)?” were considered to have a history of MI.^23^

### Covariates

Potential confounders included age (modeled as a continuous variable in regression analyses), sex, race/ethnicity, education level (below high school, high school or above),^24^ family poverty income ratio (PIR) (<1.3, 1.3–3.49, ≥3.5).^25^ marital status (married vs. unmarried);^24^ and clinical and lifestyle factors, including hypertension,^26^ diabetes,^27^ smoking status,^25^ alcohol consumption,^28^ physical activity,^27^ and body mass index (BMI, continuous). Hypertension was defined as an affirmative response to a physician diagnosis, use of antihypertensive medications, or measured blood pressure ≥140/90 mmHg. Diabetes was defined based on self-reported diagnosis, use of insulin or oral hypoglycemic agents, or laboratory criteria including fasting glucose ≥ 7.0 mmol/L or HbA1c ≥ 6.5%. Alcohol consumption was classified as non-drinkers (fewer than 12 drinks per year) and drinkers (12 or more drinks per year). Smoking status was classified as never smokers, current smokers, or former smokers based on responses regarding lifetime smoking of ≥100 cigarettes and current smoking behavior. Physical activity was defined as participation in vigorous activities during work or leisure that substantially increased breathing or heart rate for at least 10 consecutive minutes.

### Statistical analysis

Analyses were conducted in accordance with the complex sampling design of NHANES, incorporating stratification, clustering, and survey weighting to ensure national representativeness. The normality of continuous variables was assessed using graphical methods and descriptive summary statistics. Given the large sample size, parametric tests were applied owing to their robustness to moderate deviations from normality. Baseline characteristics were summarized by MI status and by quartiles of NHR (Q1–Q4), with categorical variables presented as counts and percentages and continuous variables as means with standard deviations. For continuous variables, survey-weighted one-way analysis of variance (ANOVA) was used, and overall effect sizes (ω^2^) were calculated. For binary comparisons by MI status, survey-weighted independent-samples t-tests were conducted, with Cohen’s d calculated as the effect size. For categorical variables, survey-weighted chi-square tests were conducted, with Cramer’s V calculated as the effect size. P values were adjusted for multiple comparisons using the false discovery rate (FDR) method. When overall differences were identified, pairwise comparisons were interpreted descriptively without formal post-hoc testing because of the exploratory nature of the analysis. Weighted logistic regression models were used to examine the associations of NHR, neutrophils, and HDL-C with MI, while accounting for NHANES’s complex multistage probability sampling design through the use of recommended survey weights. Covariates were selected a priori based on clinical relevance and prior literature, including demographic factors (age, sex, race/ethnicity, education, and family poverty income ratio), lifestyle factors (smoking, alcohol consumption, and physical activity), marital status, body mass index (BMI, continuous), and comorbidities (hypertension and diabetes). A sequential multivariable adjustment strategy was applied: Model 1 was unadjusted; Model 2 was adjusted for sex, age, race/ethnicity, education, and family poverty income ratio; and Model 3 was further adjusted for lifestyle factors, BMI, marital status, and comorbidities. Potential non-linear associations between NHR and MI were assessed using restricted cubic splines (RCS), with knots placed at the 25th, 50th, and 75th percentiles. Threshold analysis was performed using a segmented regression approach to identify the NHR value at which the association with MI differed below and above the threshold. Subgroup analyses were conducted across subgroups defined by age (<60 vs ≥60 years), sex, race/ethnicity, smoking status, and physical activity. Interaction tests were performed for each subgroup variable separately, and all subgroup analyses were considered exploratory given the multiple comparisons. In addition, sensitivity analyses using multiple imputation to address missing covariates were conducted to evaluate the robustness of the primary findings. All statistical analyses were performed using R software (version 4.2.1). A two-sided P value < 0.05 was considered statistically significant.

## Results

### Baseline characteristics of the study population

A total of 25,248 participants were included, among whom 1,073 had a history of MI. Compared with participants without MI, those with MI were older and had lower poverty income ratios. They also had higher BMI and higher NHR. Significant differences were also observed in race, education, gender, smoking, physical activity, diabetes, and hypertension, while alcohol consumption and marital status showed minimal variation. When participants were stratified by NHR quartiles, BMI progressively increased, while age and PIR progressively decreased across quartiles. The prevalence of MI, male sex, diabetes, smoking, and hypertension increased progressively across higher NHR quartiles, while race and education distributions shifted moderately. Alcohol consumption and marital status varied minimally across quartiles. Collectively, higher NHR was associated with male predominance, adverse cardiometabolic profiles, unhealthy lifestyle patterns, and increased MI prevalence, which reflects both cross-sectional differences by MI status and graded associations across NHR quartiles (Tables 1 - 2).

**Table 1:** – Characteristics of participants according to myocardial infarction

Characteristics	MI (Yes) n=1073	MI (No) n=24175	p value	Effect size
**Age,(years)**	66.84 (12.27)	48.84 (17.51)	<0.001	-1.190
**PIR**	2.12 (1.50)	2.56 (1.63)	<0.001	0.278
**BMI,(kg/m**^**2**^**)**	30.23 (7.06)	29.12 (6.90)	<0.001	-0.159
**NHR**	4.04(2.16)	3.44(2.16)	<0.001	-0.275
**Race, n(%)**			<0.001	0.064
Mexican American	98 ( 9.10)	3825 (15.80)		
Other Hispanic	83 ( 7.70)	2234 ( 9.20)		
Non-Hispanic White	641 (59.70)	10949 (45.30)		
Non-Hispanic Black	198 (18.50)	4915 (20.30)		
Other Race	53 ( 4.90)	2252 ( 9.30)		
**Education, n(%)**			<0.001	0.052
Below high school	375 (34.90)	5763 (23.80)		
High School or above	698 (65.10)	18412 (76.20)		
**Gender, n(%)**			<0.001	0.069
Male	713 (66.40)	11935 (49.40)		
Female	360 (33.60)	12240 (50.60)		
**Smoke, n(%)**			<0.001	0.099
Smoker	262 (24.40)	5064 (20.90)		
Smoked	462 (43.10)	5809 (24.00)		
No-smoke	349 (32.50)	13302 (55.00)		
**Physical activity, n(%)**			<0.001	0.071
High	361 (33.60)	12395 (51.30)		
Low	712 (66.40)	11780 (48.70)		
**Diabetes, n(%)**			<0.001	0.134
Yes	443 (41.30)	3897 (16.10)		
No	630 (58.70)	20278 (83.90)		
**Drink, n(%)**			0.715	0.003
Yes	778 (72.50)	17393 (71.90)		
No	295 (27.50)	6782 (28.10)		
**Marital, n(%)**			0.003	0.019
Yes	597 (55.60)	14548 (60.20)		
No	476 (44.40)	9627 (39.80)		
**Hypertension, n(%)**			<0.001	0.152
Yes	841 (78.40)	9915 (41.00)		
No	232 (21.60)	14260 (59.00)		

MI: myocardial infarction; NHR: neutrophil-to-high-density lipoprotein cholesterol ratio; HDL-C: high-density lipoprotein cholesterol; PIR: poverty income ratio; BMI: body mass index; OR: odds ratio; CI: confidence interval.

**Table 2:** – Characteristics of participants according to NHR quartiles

Characteristics	NHR				p value	Effect size
	Q1(n=6312)	Q2(n=6312)	Q3(n=6312)	Q4(n=6312)		
**Age,mean(SD)**	50.74 (17.68)	50.21 (17.89)	49.36 (17.87)	48.12 (17.24)	<0.001	0.003
**PIR,mean(SD)**	2.77 (1.66)	2.64 (1.64)	2.47 (1.61)	2.27 (1.56)	<0.001	0.013
**BMI(kg/m**^**2**^**),mean(SD)**	26.70 (5.87)	28.41 (6.31)	29.71 (6.61)	31.85 (7.67)	<0.001	0.074
**MI, n(%)**					<0.001	0.061
Yes	170 (2.70)	247 (3.90)	271 (4.30)	385 (6.10)		
No	6142 (97.30)	6065 (96.10)	6041 (95.70)	5927 (93.90)		
**Gender, n(%)**					<0.001	0.159
Male	2458 (38.90)	2995 (47.40)	3368 (53.40)	3827 (60.60)		
Female	3854 (61.10)	3317 (52.60)	2944 (46.60)	2485 (39.40)		
**Race, n(%)**					<0.001	0.133
Mexican American	632 (10.00)	904 (14.30)	1199 (19.00)	1188 (18.80)		
Non-Hispanic Black	2148 (34.00)	1308 (20.70)	947 (15.00)	710 (11.20)		
Non-Hispanic White	2450 (38.80)	2870 (45.50)	2983 (47.30)	3287 (52.10)		
Other Hispanic	448 (7.10)	602 (9.50)	649 (10.30)	618 (9.80)		
Other Race	634 (10.00)	628 (9.90)	534 (8.5)0)	509 (8.10)		
**Education, n(%)**					<0.001	0.071
Below high school	1273 (20.20)	1464 (23.20)	1604 (25.40)	1797 (28.50)		
High School or above	5039 (79.80)	4848 (76.80)	4708 (74.60)	4515 (71.50)		
**Smoke, n(%)**					<0.001	0.120
Smoker	904 (14.30)	1086 (17.20)	1337 (21.20)	1999 (31.70)		
Smoked	1525 (24.20)	1569 (24.90)	1637 (25.90)	1540 (24.40)		
No-smoke	3883 (61.50)	3657 (57.90)	3338 (52.90)	2773 (43.90)		
**Physical activity, n(%)**					<0.001	0.103
High	3588 (56.80)	3340 (52.90)	3126 (49.50)	2702 (42.80)		
Low	2724 (43.20)	2972 (47.10)	3186 (50.50)	3610 (57.20)		
**Diabetes, n(%)**					<0.001	0.137
Yes	667 (10.60)	947 (15.00)	1170 (18.50)	1556 (24.70)		
No	5645 (89.40)	5365 (85.00)	5142 (81.50)	4756 (75.30)		
**Drink, n(%)**					<0.001	0.025
Yes	4448 (70.5)	4525 (71.7)	4550 (72.1)	4648 (73.6)		
No	1864 (29.5)	1787 (28.3)	1762 (27.9)	1664 (26.4)		
**Marital, n(%)**					<0.001	0.041
Yes	3583 (56.80)	3794 (60.10)	3849 (61.00)	3919 (62.10)		
No	2729 (43.20)	2518 (39.90)	2463 (39.00)	2393 (37.90)		
**Hypertension, n(%)**					<0.001	0.055
Yes	2482 (39.30)	2625 (41.60)	2692 (42.60)	2957 (46.80)		
No	3830 (60.70)	3687 (58.40)	3620 (57.40)	3355 (53.20)		

MI: myocardial infarction; NHR: neutrophil-to-high-density lipoprotein cholesterol ratio; HDL-C: high-density lipoprotein cholesterol; PIR: poverty income ratio; BMI: body mass index; OR: odds ratio; CI: confidence interval.

### Association between NHR, Neutrophils, HDL-C, and MI

Higher NHR was consistently associated with higher odds of MI across unadjusted and partially adjusted models (Models 1 and 2). After full adjustment for demographic and clinical covariates (Model 3), participants in the highest quartile of NHR had 49% higher odds of MI compared with those in the lowest quartile, with a significant dose–response trend across quartiles. For the neutrophil count, the association with MI was attenuated in the fully adjusted model. Participants in the highest quartile had a non-significant 11% higher odds of MI compared with the lowest quartile. The trend across quartiles was not statistically significant. For HDL-C, higher levels were consistently associated with lower odds of MI. In the fully adjusted model, participants in the highest quartile had 38% lower odds of MI compared with the lowest quartile and exhibited a significant dose–response trend across quartiles. These results indicate that NHR showed more consistent associations with MI than neutrophil count or HDL-C alone (Table 3).

**Table 3:** – Logistic regression analysis of NHR, neutrophils, and HDL-C with MI

	Model 1	Model 2	Model 3
**NHR Inter quartile**	**OR(95%CI) p-value**	**OR(95%CI) p-value**	**OR(95%CI) p-value**
Q1 < 2.139	Ref	Ref	Ref
Q2 2.139~3.067	1.52(1.18. 1.96) 0.001	1.46(1.11. 1.920 008	1.26(0.95. 1.67) 0.105
Q3 3.067~4.308	1.70(1.35. 2.14) <0.001	1.64(1.29. 2.08) <0.001	1.27(0.99. 1.61) 0.052
Q4 >4.308	2.38(1.85. 3.06) <0.001	2.39(1.82. 3.14) <0.001	1.49(1.11.2.00) 0.008
P for trend	<0.001	<0.001	0.007
**Neutrophils Inter quartile**			
Q1 < 3.1	Ref	Ref	Ref
Q2 3.1~4.0	1.13 (0.87. 1.46) 0.353	1.05 (0.81. 1.36) 0.706	0.91 (0.70. 1.19) 0.497
Q3 4.0~5.1	1.52 (1.20. 1.94) <0.001	1.34 (1.04. 1.72) 0.025	1.07 (0.83. 1.38) 0.602
Q4 >5.1	1.74 (1.39. 2.19) <0.001	1.66 (1.31. 2.09) <0.001	1.11 (0.87. 1.43) 0.397
P for trend	<0.001	<0.001	0.178
**HDL-C Inter quartile**			
Q1 < 1.06	Ref	Ref	Ref
Q2 1.06~1.29	0.75 (0.60. 0.93) 0.010	0.71 (0.56. 0.90) 0.005	0.80 (0.63. 1.01) 0.060
Q3 1.29~1.60	0.49 (0.40. 0.61) <0.001	0.49 (0.39. 0.62) <0.001	0.61 (0.48. 0.77) <0.001
Q4 >1.60	0.49 (0.38. 0.63) <0.001	0.45 (0.35. 0.59) <0.001	0.62 (0.47. 0.81) <0.001
P for trend	<0.001	<0.001	<0.001

Model 1 was unadjusted. Model 2 adjusted for sex, age, race, family poverty income ratio, and education level. Model 3 adjusted for all covariates. MI: myocardial infarction; NHR: neutrophil-to-high-density lipoprotein cholesterol ratio; HDL-C: high-density lipoprotein cholesterol; BMI: body mass index; OR: odds ratio; CI: confidence interval.

RCS analysis was also performed to evaluate the potential nonlinear association between NHR and MI. As shown in Figure 2, after adjustment for all covariates, both the overall association and the nonlinear component were statistically significant, suggesting a modest nonlinear relationship between NHR and MI. Threshold analysis identified a data-driven inflection point at an NHR of 6.93. Below this point, a higher NHR was associated with higher odds of MI, whereas the association above the inflection point was not statistically significant. However, this threshold finding should be interpreted cautiously as exploratory and descriptive rather than as evidence of a clinically meaningful cutoff, particularly given the relatively limited number of participants in the upper tail of the NHR distribution. As shown in Table 4, the likelihood ratio test supported the threshold model.

**Figure 2:** – RCS curves illustrating the adjusted association between NHR and myocardial infarction odds in the full cohort (n = 25,248), with three knots. The solid line represents the odds ratio (OR), and the dashed lines indicate 95% confidence intervals. NHR: neutrophil-to-high-density lipoprotein cholesterol ratio; MI: myocardial infarction; OR: odds ratio; CI: confidence interval.

**Table 4:** – Threshold analysis of NHR and myocardial infarction odds

	OR(95%CI)	p-value
Inflection point	6.93	
NHR < 6.93	1.10 (1.05. 1.15)	<0.001
NHR ≥ 6.93	0.99 (0.92. 1.03)	0.717
Log likelihood ratio		<0.001

### Subgroup analysis

Additional subgroup analyses suggested that the association between NHR and MI may vary across subgroups. As shown in Figure 3, positive associations between NHR and MI were observed in several subgroups, including participants aged ≥60 years, women, those with BMI >25 kg/m^2^, those with low physical activity, those with hypertension, those with diabetes, and those without a history of stroke. However, given the exploratory nature of these analyses and the multiple interaction tests performed, the subgroup findings should be interpreted cautiously. Among the examined subgroups, the interaction by sex was statistically significant, suggesting that the association may be stronger in women.

**Figure 3:** – Subgroup analysis of the association between NHR and MI. Odds ratios and 95% confidence intervals are shown for each predefined subgroup. Component-specific P values indicate the statistical significance of the association between NHR and MI within each subgroup, while P values for interaction assess potential effect modification across subgroups. NHR: neutrophil-to-high-density lipoprotein cholesterol ratio; MI: myocardial infarction; OR: odds ratio; CI: confidence interval.

### Sensitivity analysis

As part of the sensitivity analyses, we used multiple imputation by chained equations to impute missing covariates while retaining the observed NHR and MI values and incorporating the NHANES survey design and sampling weights into each imputed dataset. The results were materially consistent with the findings of the primary complete-case analysis (Supplementary Table 2). In the fully adjusted model, higher NHR remained associated with higher odds of MI. Participants in the highest quartile had significantly higher odds of MI than those in the lowest quartile (OR 1.536, 95% CI 1.141–2.067; P=0.005), and the trend across quartiles was also statistically significant P=0.004). In contrast, neutrophil count alone was not significantly associated with MI after full adjustment, whereas higher HDL-C levels remained inversely associated with MI. These findings support the robustness of the primary findings.

A schematic overview of the experimental design and major findings is presented in the Central Illustration.

## Discussion

In this cross-sectional analysis of 25,248 NHANES participants, higher NHR levels were associated with higher odds of MI, thus reflecting prevalent rather than incident disease. After multivariable adjustment, participants in the highest NHR quartile had significantly higher odds of MI than those in the lowest quartile, whereas neutrophil count alone was not significantly associated with MI, and HDL-C levels were inversely associated with MI. Restricted cubic spline analysis suggested a modest nonlinear association between NHR and MI, with a data-driven inflection point at an NHR of approximately 6.93. Because most participants had NHR values below this point and relatively few were in the upper tail, the observed nonlinearity should be interpreted cautiously as exploratory and descriptive rather than evidence of a clinically meaningful cutoff. Subgroup analyses suggested that the association may be stronger in women; however, these findings should be interpreted as exploratory. Sensitivity analyses also yielded materially consistent results, supporting the robustness of the primary findings. Overall, our results support an association between higher NHR and higher odds of MI in this population.

Our results align with and extend existing evidence regarding the clinical relevance of NHR. Community-based cohort data from Taiwan indicate that NHR outperforms NLR in predicting cardiovascular disease risk.^29^ Clinical studies in CAD patients have associated higher NHR with more severe coronary stenosis, as reflected by Gensini scores (AUC~0.73).^30^ In patients with type 2 diabetes and acute coronary syndrome, NHR has also demonstrated strong predictive and diagnostic value.^13^ Similarly, a Chinese cohort study found that elevated NHR predicted adverse cardiovascular outcomes among pre-diabetic individuals.^31^ More recently, NHANES data revealed that elevated NHR, combined with cognitive impairment, was associated with substantially higher all-cause mortality in older adults.^32^ Unlike prior studies largely conducted in clinical or high-risk populations, our study utilizes a nationally representative sample and identifies a robust association between NHR and myocardial infarction in the general U.S. adult population, highlighting its potential epidemiological relevance. Traditional markers such as LDL-C and hsCRP reflect lipid burden and systemic inflammation separately, whereas NHR integrates innate inflammatory activity and an anti-atherogenic lipid fraction into a single index, capturing the balance between pro-inflammatory and protective pathways and potentially explaining its relevance in population-based studies.

Accumulating evidence suggests that NHR may be associated with myocardial infarction through mechanisms involving inflammation, dysregulated lipid metabolism, and vascular endothelial dysfunction.^33,34^

Neutrophils are among the first immune cells to infiltrate the infarcted myocardium and reach peak levels within 24 hours after MI Upon infiltrating the infarct area, neutrophils release calprotectin (S100A8/A9) complexes, which activate Toll-like receptor 4 and NOD-like receptor inflammasomes, thereby promoting the secretion of pro-inflammatory cytokines such as interleukin-1β. These cytokines stimulate bone marrow hematopoiesis, thereby sustaining neutrophil production and amplifying inflammatory responses, which in turn exacerbate myocardial injury.^35,36^ Activated neutrophils also release proteases and pro-inflammatory mediators that polarize macrophages toward a pro-inflammatory (M1-like) phenotype, thereby inhibiting tissue repair.^37^ Studies have reported that in ST-elevation myocardial infarction, neutrophils and other inflammatory cells adhere to and transmigrate through endothelial cells via integrins. During this process, they release pro-inflammatory cytokines that disrupt endothelial function, exacerbate inflammation and myocardial injury, aggravate ischemia–reperfusion injury, and promote ventricular remodeling.^4^

Conversely, HDL-C exerts cardioprotective effects through its bioactive molecules by activating cellular signaling pathways and inhibiting mitochondrial permeability transition pore opening, which in turn reduces cardiomyocyte apoptosis and necrosis. Additionally, HDL-C suppresses NF-κB activation, thereby reducing the release of inflammatory cytokines and attenuating myocardial inflammation.^38^ HDL-C also facilitates reverse cholesterol transport, clearing cholesterol from arterial plaques and thereby exerting protective effects against myocardial infarction.^39^

Subgroup analyses suggested that the association between NHR and MI may be stronger in women than in men; however, these findings should be interpreted cautiously because the subgroup analyses were exploratory and involved multiple comparisons and interaction tests. Prior evidence indicates that women with acute myocardial infarction (AMI) may exhibit plaque characteristics distinct from those of men, with a greater propensity for plaque erosion than for plaque rupture. Approximately 30% of women with AMI have non-obstructive coronary artery disease on angiography, a proportion higher than that observed in men, which may be related to microvascular dysfunction or coronary artery spasm.^40^ Furthermore, estrogen is thought to confer relative protection against cardiovascular disease in premenopausal women; this protection diminishes after menopause, with cardiovascular risk approaching that of men.^41^ In participants with stroke, the association between NHR and MI was not statistically significant; however, this finding should not be overinterpreted. Prior studies suggest that acute ischemic stroke may trigger stress-related and immunologic responses that increase circulating neutrophil levels.^42^ In addition, stroke-associated neuroimmune activation may alter peripheral inflammatory profiles and thereby affect the specificity of NHR in this subgroup.^43^ Overall, these subgroup findings should be regarded as hypothesis-generating rather than evidence of definitive biological effect modification.

The robustness of our findings was strengthened by the large, nationally representative NHANES sample and adjustment for a broad range of potential confounders. This study suggests that NHR may serve as a biomarker associated with MI, with subgroup analyses indicating that the association may be stronger in women. However, the cross-sectional design and reliance on self-reported MI history preclude causal inference, limit the assessment of temporality, and preclude differentiation between recent and remote MI events. In addition, because NHR was measured at the time of the NHANES examination rather than before the occurrence of MI, it may have been influenced by prior MI itself, as well as by subsequent treatment and behavioral changes after MI, including lipid-lowering therapy and lifestyle modification. Therefore, reverse causation cannot be excluded. Selection bias may also have resulted from the exclusion of participants with missing data, and residual confounding cannot be fully ruled out. In particular, we were unable to consistently adjust for lipid-lowering therapy and established lipid and inflammatory biomarkers, such as LDL-C, triglycerides, and hsCRP, across all included NHANES cycles. These factors may have influenced both NHR and MI status and thus remain potential sources of residual confounding. Although E-value analyses suggested that relatively strong unmeasured confounding would be required to fully account for the observed association, some adjusted variables may lie on the causal pathway, raising the possibility of over-adjustment. Finally, predictive performance was not formally evaluated, and the generalizability of these findings beyond the US adult population may be limited.

## Conclusion

In summary, our findings indicate that higher neutrophil-to-HDL cholesterol ratio levels are associated with increased odds of myocardial infarction, with a modest non-linear pattern observed across the NHR range. These associations persisted after multivariable adjustment and reflect myocardial infarction prevalence rather than the risk of incident events. Subgroup analyses suggested that the association may be stronger in females; however, these results should be interpreted as exploratory. Given the cross-sectional design, causality cannot be inferred, and prospective studies are needed to clarify temporal relationships and further elucidate the underlying mechanisms.

## * Supplementary Materials

Supplementary material 1 For Supplementary Table 1, please click here.https://abccardiol.org/supplementary-material/2026/12308/2025-0718_AO_Supplementary_Table1.pdf

Supplementary material For Supplementary Table 2, please click here. https://abccardiol.org/supplementary-material/2026/12308/2025-0718_AO_Supplementary_Table2.pdf
